# Caesarean section and risk of type 1 diabetes

**DOI:** 10.1007/s00125-024-06176-7

**Published:** 2024-05-31

**Authors:** Tarini Singh, Andreas Weiss, Kendra Vehik, Jeffrey Krischer, Marian Rewers, Jorma Toppari, Åke Lernmark, William Hagopian, Beena Akolkar, Ezio Bonifacio, Anette-G. Ziegler, Christiane Winkler, TEDDY Study Group

**Affiliations:** 1 Institute of Diabetes Research, Helmholtz Munich, German Research Center for Environmental Health, Munich, Germany; 2 Health Informatics Institute, Morsani College of Medicine, University of South Florida, Tampa, FL USA; 3 Barbara Davis Center for Childhood Diabetes, University of Colorado, Aurora, CO USA; 4 Department of Pediatrics, Turku University Hospital, Turku, Finland; 5 Institute of Biomedicine, Research Centre for Integrative Physiology and Pharmacology, University of Turku, Turku, Finland; 6 Centre for Population Health Research, University of Turku, Turku, Finland; 7 Department of Clinical Sciences, Lund University/CRC, Skåne University Hospital SUS, Malmo, Sweden; 8 Pacific Northwest Research Institute, Seattle, WA USA; 9 National Institute of Diabetes and Digestive and Kidney Diseases, Bethesda, MD USA; 10 Technische Universität Dresden, Center for Regenerative Therapies Dresden, Dresden, Germany; 11 Paul Langerhans Institute Dresden of Helmholtz Munich, University Hospital Carl Gustav Carus and Faculty of Medicine, Technische Universität Dresden, Dresden, Germany; 12 Institute for Diabetes and Obesity, Helmholtz Munich, German Research Center for Environmental Health, Munich, Germany; 13 Forschergruppe Diabetes at Klinikum rechts der Isar, School of Medicine, Technical University Munich, Munich, Germany; 14 Forschergruppe Diabetes e.V. at Helmholtz Munich, German Research Center for Environmental Health, Munich, Germany

**Keywords:** Caesarean section, Progression, Type 1 diabetes, Type 1 diabetes susceptibility genes

## Abstract

**Aims/hypothesis:**

Delivery by Caesarean section continues to rise globally and has been associated with the risk of developing type 1 diabetes and the rate of progression from pre-symptomatic stage 1 or 2 type 1 diabetes to symptomatic stage 3 disease. The aim of this study was to examine the association between Caesarean delivery and progression to stage 3 type 1 diabetes in children with pre-symptomatic early-stage type 1 diabetes.

**Methods:**

Caesarean section was examined in 8135 children from the TEDDY study who had an increased genetic risk for type 1 diabetes and were followed from birth for the development of islet autoantibodies and type 1 diabetes.

**Results:**

The likelihood of delivery by Caesarean section was higher in children born to mothers with type 1 diabetes (adjusted OR 4.61, 95% CI 3.60, 5.90, *p*<0.0001), in non-singleton births (adjusted OR 4.35, 95% CI 3.21, 5.88, *p*<0.0001), in premature births (adjusted OR 1.91, 95% CI 1.53, 2.39, *p*<0.0001), in children born in the USA (adjusted OR 2.71, 95% CI 2.43, 3.02, *p*<0.0001) and in children born to older mothers (age group >28–33 years: adjusted OR 1.19, 95% CI 1.04, 1.35, *p*=0.01; age group >33 years: adjusted OR 1.80, 95% CI 1.58, 2.06, *p*<0.0001). Caesarean section was not associated with an increased risk of developing pre-symptomatic early-stage type 1 diabetes (risk by age 10 years 5.7% [95% CI 4.6%, 6.7%] for Caesarean delivery vs 6.6% [95% CI 6.0%, 7.3%] for vaginal delivery, *p*=0.07). Delivery by Caesarean section was associated with a modestly increased rate of progression to stage 3 type 1 diabetes in children who had developed multiple islet autoantibody-positive pre-symptomatic early-stage type 1 diabetes (adjusted HR 1.36, 95% CI 1.03, 1.79, *p*=0.02). No interaction was observed between Caesarean section and non-HLA SNPs conferring susceptibility for type 1 diabetes.

**Conclusions/interpretation:**

Caesarean section increased the rate of progression to stage 3 type 1 diabetes in children with pre-symptomatic early-stage type 1 diabetes.

**Data availability:**

Data from the TEDDY study (https://doi.org/10.58020/y3jk-x087) reported here will be made available for request at the National Institute of Diabetes and Digestive and Kidney Diseases (NIDDK) Central Repository (NIDDK-CR) Resources for Research (R4R) (https://repository.niddk.nih.gov/).

**Graphical Abstract:**

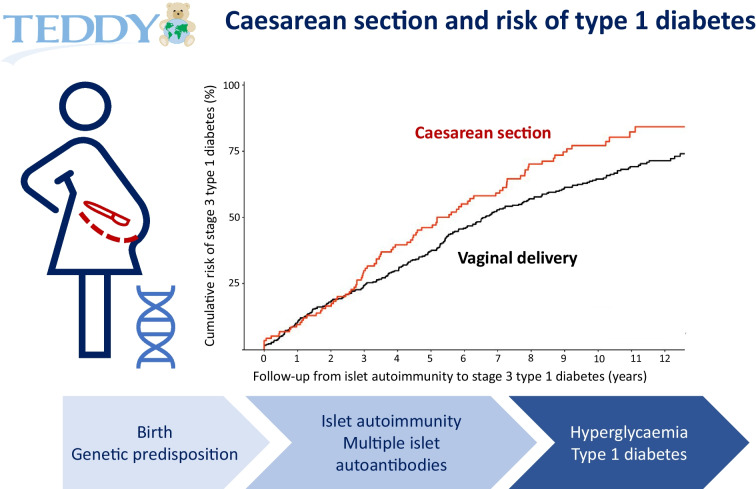

**Supplementary Information:**

The online version contains peer-reviewed but unedited supplementary material available at 10.1007/s00125-024-06176-7.


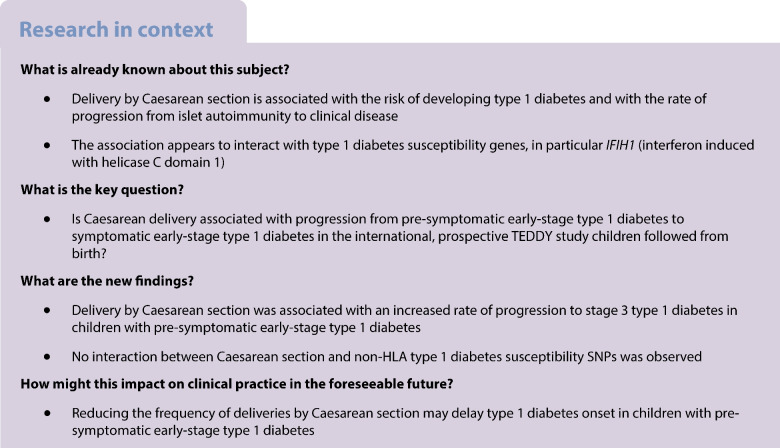


## Introduction

The incidence of type 1 diabetes is increasing [1]. Concurrently, the prevalence of Caesarean section deliveries has increased and there is an association with the incidence rate of type 1 diabetes [2]. A meta-analysis revealed a 20% increase in the risk of childhood-onset type 1 diabetes in children delivered by Caesarean section [3]. A previous prospective birth cohort study indicated that Caesarean section accelerates disease progression but has no impact on the incidence of islet autoimmunity [4]. In that study, Caesarean section appeared to interact with type 1 diabetes susceptibility genes, in particular the *IFIH1* gene (interferon induced with helicase C domain 1), suggesting modulation of the response to a diabetes-relevant environment by both Caesarean section and *IFIH1.* Here, the aim was to validate and extend these findings in the international TEDDY study including over 8000 prospectively followed children with type 1 diabetes-susceptible HLA genotypes, a group representing approximately 50% of children with type 1 diabetes.

## Methods

### Study cohort

The TEDDY study is a prospective cohort study of 8676 children with an increased genetic risk for type 1 diabetes. The study includes six clinical research centres in the USA (Colorado, Georgia/Florida, Washington), Finland, Germany and Sweden. The detailed study design and methods have been previously published [5] (see electronic supplementary material [ESM] Methods]). Data on mode of delivery, maternal diabetes status, maternal age, singleton birth, gestational age and birthweight were retrospectively collected either by structured questionnaires or through interviews at enrolment (age 3 months).

### SNPs

SNPs for *IFIH1* (rs1990760), *MIR3681HG* (rs1534422), *CTSH* (rs3825932) and *TNFAIP3* (rs2327832) were genotyped using the Illumina ImmunoChip (USA) [6].

### Study outcome

Islet autoantibodies (IAA, GADA and IA-2A) were measured every 3 months for the first 4 years and biannually thereafter [5]. Date of persistent autoimmunity was defined as the draw date of the first sample of two consecutive samples that deemed a child as being persistent confirmed positive for a specific autoantibody. The presence of persistent multiple islet autoantibodies (pre-symptomatic early-stage type 1 diabetes) was defined as the presence of at least two persistent and confirmed islet autoantibodies. Stage 3 type 1 diabetes was diagnosed according to ADA criteria [7].

### Statistical analyses

Logistic regression was used to identify factors associated with Caesarean section. Kaplan–Meier analysis was used to calculate risk and the logrank test was used to compare outcome probabilities by delivery mode. A Cox proportional hazards model for risk of progression to stage 3 type 1 diabetes from pre-symptomatic early-stage type 1 diabetes was used to determine HRs for multiple covariates. Evidence for interaction between Caesarean section and type 1 diabetes susceptibility genes associated with type 1 diabetes was investigated using a Cox proportional hazards model and by Kaplan–Meier analysis after stratification for genotype. Children with missing data were excluded from the relevant analyses. Two-tailed *p* values <0.05 were considered significant. Analyses were performed using R version 4.3.0 (https://www.R-project.org/, accessed 6 June 2023).

## Results

**Fig. 1:**
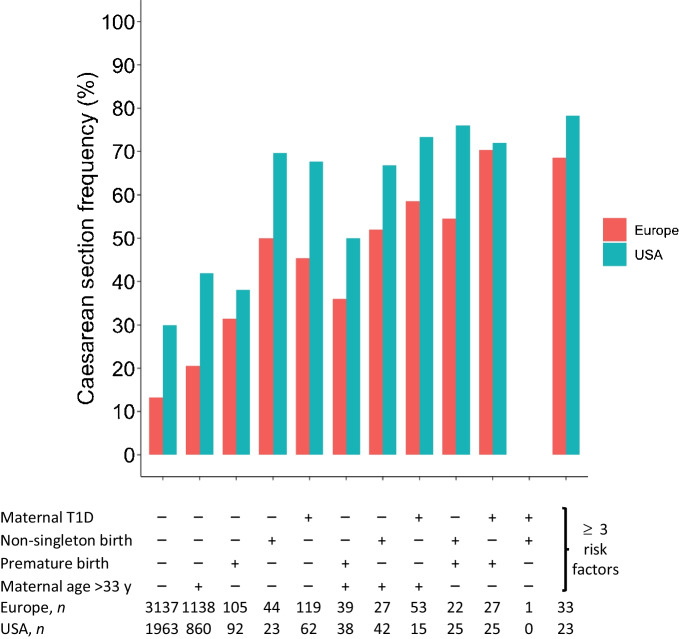
Frequency of Caesarean section by different birth factors. T1D, type 1 diabetes; y, years

The mode of delivery and SNP genotyping data were available for 8135 children. Of these, 2110 (25.9%) were delivered by Caesarean section and 6025 (74.1%) by vaginal delivery. The likelihood of delivery by Caesarean section was higher in children born to mothers with type 1 diabetes than in children born to mothers without type 1 diabetes (adjusted OR 4.61, 95% CI 3.60, 5.90, *p*<0.0001), in non-singleton births (adjusted OR 4.35, 95% CI 3.21, 5.88, *p*<0.0001), in births at <37 weeks of gestation (adjusted OR 1.91, 95% CI 1.53, 2.39, *p*<0.0001), in children born in the USA (adjusted OR 2.71, 95% CI 2.43, 3.02, *p*<0.0001) and in children born to older mothers (>28–33 years: adjusted OR 1.19, 95% CI 1.04, 1.35, *p*=0.01; >33 years: adjusted OR 1.80, 95% CI 1.58, 2.06, *p*<0.0001; ESM Table 1). The frequency of Caesarean section births was increased in non-singleton births, in children born prematurely, in children born in the USA and in children born to mothers with type 1 diabetes irrespective of other risk factors (Fig. 1).

**Fig. 2:**
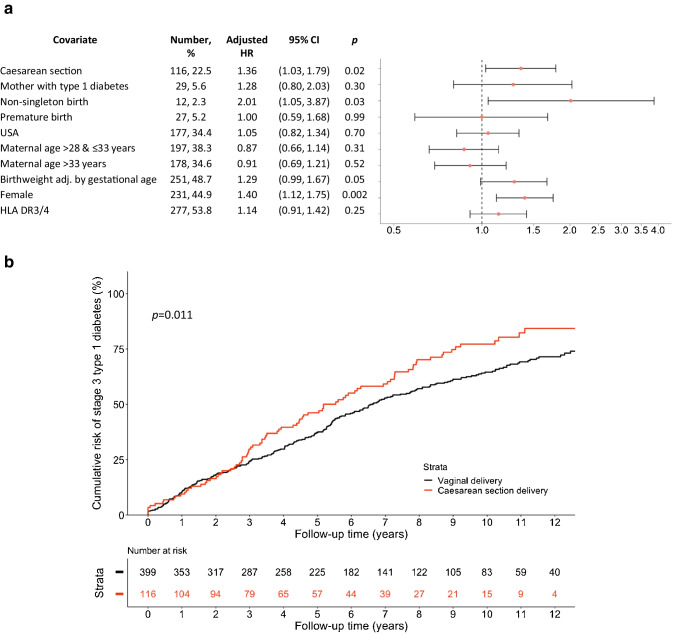
(**a**) Multivariate Cox proportional hazard model for the risk of developing stage 3 type 1 diabetes in children with pre-symptomatic early-stage type 1 diabetes (multiple islet autoantibodies). (**b**) Caesarean section and risk for progression to stage 3 type 1 diabetes from seroconversion in children with pre-symptomatic early-stage type 1 diabetes (positive for multiple islet autoantibodies). Kaplan–Meier analysis of the probability of developing stage 3 type 1 diabetes in children delivered by Caesarean section (red line) or vaginally (black line). The numbers under the graph indicate the numbers of children still under observation at each time point. *p* values were obtained from logrank tests comparing children delivered by Caesarean section with children delivered vaginally

Pre-symptomatic early-stage type 1 diabetes developed in 515 children, including 331 (64.3%) children who progressed to stage 3 type 1 diabetes. Caesarean section was not associated with an increased risk of developing pre-symptomatic early-stage type 1 diabetes (risk by age 10 years 5.7% [95% CI 4.6%, 6.7%] for Caesarean delivery vs 6.6% [95% CI 6.0%, 7.3%] for vaginal delivery, *p*=0.07; ESM Fig. 1). However, Caesarean section was associated with faster progression to stage 3 type 1 diabetes in children with pre-symptomatic early-stage type 1 diabetes (adjusted HR 1.36, 95% CI 1.03, 1.79, *p*=0.02; Fig. 2a). The association was also observed in a sensitivity analysis that excluded children born to mothers with type 1 diabetes, premature births and non-singleton births performed in the whole population (adjusted HR 1.44, 95% CI 1.07, 1.94, *p*=0.01, ESM Fig. 2a) or in the European population (adjusted HR 2.06, 95% CI 1.39, 3.07, *p*=0.0003, ESM Fig. 2b). The 10-year risk for progression to stage 3 type 1 diabetes in children with pre-symptomatic early-stage type 1 diabetes was 76.2% (95% CI 65.5%, 83.6%) for those born by Caesarean section and 64.1% (95% CI 58.2%, 69.1%) for those born by vaginal delivery (*p*=0.02; Fig. 2b). The increased rate of progression was observed from around 3 years after developing islet autoantibodies. A 50% progression rate from the first autoantibody to stage 3 type 1 diabetes occurred at 5.3 years in children born by Caesarean section and 6.7 years in children born by vaginal delivery.

Type 1 diabetes susceptibility genes have previously been found to interact with Caesarean section (*IFIH1* [4]) or to be associated with increased progression to stage 3 type 1 diabetes (*TNFAIP3*, *CTSH*, *MIR2681HG* [8]). After stratification for Caesarean section, no differences in progression to stage 3 type 1 diabetes were observed for the *IFIH1* (rs1990760) genotypes (ESM Fig. 3a). For the *MIR3681HG* (rs1534422) and *TNFAIP3* (rs2327832) genotypes, an increased risk for progression was observed only in children born by Caesarean section and with the type 1 diabetes-susceptible *MIR3681HG* GG genotype (ESM Fig. 3b) or *TNFAIP3* GG genotype (ESM Fig. 3d). However, the numbers of children in these categories were low. For *CTSH* (rs3825932), the risk for progression was lowest in children born by vaginal delivery and with the non-susceptible *CTSH* AA/AG genotype and highest in children born by Caesarean section and with the susceptible *CTSH* GG genotype (ESM Fig. 3c)*.*

## Discussion

This study demonstrates that Caesarean section delivery is associated with an accelerated progression to clinical type 1 diabetes in children with pre-symptomatic early-stage type 1 diabetes. These results confirm earlier findings in offspring of parents with type 1 diabetes [4] and underscore the potential for interventions moderating the frequency of Caesarean section to delay clinical type 1 diabetes onset.

The TEDDY study is the largest prospective birth cohort study in children, combining data from four countries. In contrast to BABYDIAB [4], the TEDDY study includes children with and without a family history of type 1 diabetes. A study limitation is that it includes only children with an increased genetic risk for type 1 diabetes. The finding that an intervention at birth has long-term effects on disease progression after the onset of islet autoimmunity in both studies is intriguing. The mechanism behind this association is unclear. Previous data suggest that Caesarean section delivery is associated with alterations in the early development of the immune system that persist beyond the perinatal period [9]. Minor differences in the gut microbiome have been reported between infants born by vaginal delivery and those born by Caesarean section delivery. However, it is unknown whether any of these and other differences persist beyond early childhood. Interaction between delivery by Caesarean section and the *IFIH1* genotype previously found in offspring of parents with type 1 diabetes or genes previously associated with progression to stage 3 type 1 diabetes was not observed in the TEDDY study. The increased rate of progression was observed from around 3 years after developing islet autoantibodies. Therefore, the delayed acceleration may be partially due to the transition from single to multiple islet autoantibodies, which in TEDDY occurred at a median time of 6.9 months.

Numerous factors associate with an increase in Caesarean section delivery, as previously reported [4] and shown in our study. The increased progression rate to stage 3 type 1 diabetes associated with Caesarean section was modest but remained after adjustment for such factors and in a sensitivity analysis that excluded children from the highest Caesarean section delivery categories. However, we cannot exclude the possibility that our findings are confounded by other factors associated with Caesarean section delivery, including maternal BMI. Moreover, we do not have stage data to assess whether Caesarean section was associated with a reduced time in stage 1, stage 2 or both stages of type 1 diabetes. The frequency of births by Caesarean section have increased substantially in recent decades [2]. While maternal and child safety is a major consideration for opting for Caesarean section delivery, among the large number of full-term, singleton-birth children of young mothers without type 1 diabetes in the TEDDY study, it is noteworthy that 13% of European children and 30% of children in the USA were delivered by Caesarean section. Some Caesarean section deliveries are reported to be voluntary or unnecessary [10].

In summary, these findings demonstrate that delivery by Caesarean section is associated with an accelerated progression to stage 3 type 1 diabetes in children with pre-symptomatic early-stage type 1 diabetes. The balance between the advantages and the disadvantages of Caesarean section delivery should be considered, especially in type 1 diabetes at-risk groups.

### Supplementary Information

Below is the link to the electronic supplementary material.

Supplementary file1 (PDF 838 KB)

## Data Availability

Data from the TEDDY study (10.58020/y3jk-x087 [11]) reported here will be made available for request at the National Institute of Diabetes and Digestive and Kidney Diseases (NIDDK) Central Repository (NIDDK-CR) Resources for Research (R4R) (https://repository.niddk.nih.gov/).
