# Supplementary material for: Caesarean section and risk of type 1 diabetes
Source: Diabetologia. 2024 May 31;67(8):1582–7. doi: 10.1007/s00125-024-06176-7 (PMC11343945; doi:10.1007/s00125-024-06176-7)
Supplement: Supplementary file 1 — Supplementary file1 (PDF 838 KB) [file 125_2024_6176_MOESM1_ESM.pdf]

Supplementary information: Caesarean section and risk of type 1 diabetes

ESM Methods

TEDDY Study Design

TEDDY is a prospective cohort study with the primary objective of identifying environmental factors associated with increased risk of islet autoimmunity and type 1 diabetes; it includes three centres in the USA(Colorado, Georgia/Florida and Washington) and three in Europe (Finland, Germany and Sweden). Infants younger than 4.5 months and carrying type-1-diabetes-associated HLA alleles (HLA-DR, DQ) were eligible to participate. There were no exclusions in the screening phase. All infants screened by TEDDY were eligible at each clinical center regardless of race/ethnicity or gender. Both girls and boys, and members of all racial and ethnic populations were enrolled and appropriately represent each geographic region of the study. There were no exclusions based on gender, racial or ethnic group. From 2004 to 2010, TEDDY screened >420,000 newborns and identified 21,589 children with high-risk HLA-DR/DQ genotypes. Of these, 8,676 (932 with first-degree family history of type 1 diabetes and 7,744 without such history) were enrolled in the prospective follow-up. Participants were seen and blood collected every 3 months up to 4 years of age, and every 6 months thereafter. Written informed consent was obtained from the parents. The study was approved by the ethical committees of the participating sites.

The Environmental Determinants of Diabetes in the Young (TEDDY) study (2007): study design. Pediatr Diabetes;8:286-298.

ESM Results

ESM Table 1: Association of Caesarean section with pregnancy and birth factors (adjusted for the covariables)

	Odds ratios	95%CI	p value
**Mother with type 1 diabetes**	4.61	(3.60, 5.90)	<0.0001
**Birth weight adjusted by gestational age**	1.22	(1.09, 1.38)	0.0007
**Region**			
**Europe**			
**USA**	2.71	(2.43, 3.02)	<0.0001
**HLA**			
**DR3/4**	0.97	(0.87, 1.09)	0.642
**Others**			
**Sex**			
**male**			
**female**	0.99	(0.89, 1.10)	0.835
**Premature birth**	1.91	(1.53, 2.39)	<0.0001
**Non-singleton birth**	4.35	(3.21, 5.88)	<0.0001
**Maternal age**			
**≤ 28 years**			
**> 28 ≤ 33 years**	1.19	(1.04, 1.35)	0.01
**> 33 years**	1.80	(1.58, 2.06)	<0.0001

ESM Figure 1: Caesarean section and risk for pre-symptomatic early-stage type 1 diabetes (multiple islet autoantibodies). Kaplan-Meier analysis for the probability of pre-symptomatic early-stage type 1 diabetes in children delivered by Caesarean section (red line) or vaginally (black line). Numbers under the abscissa are the number of children still followed at each time point. *P* values are derived from log-rank tests comparing children delivered by Caesarean section with children born by vaginal delivery

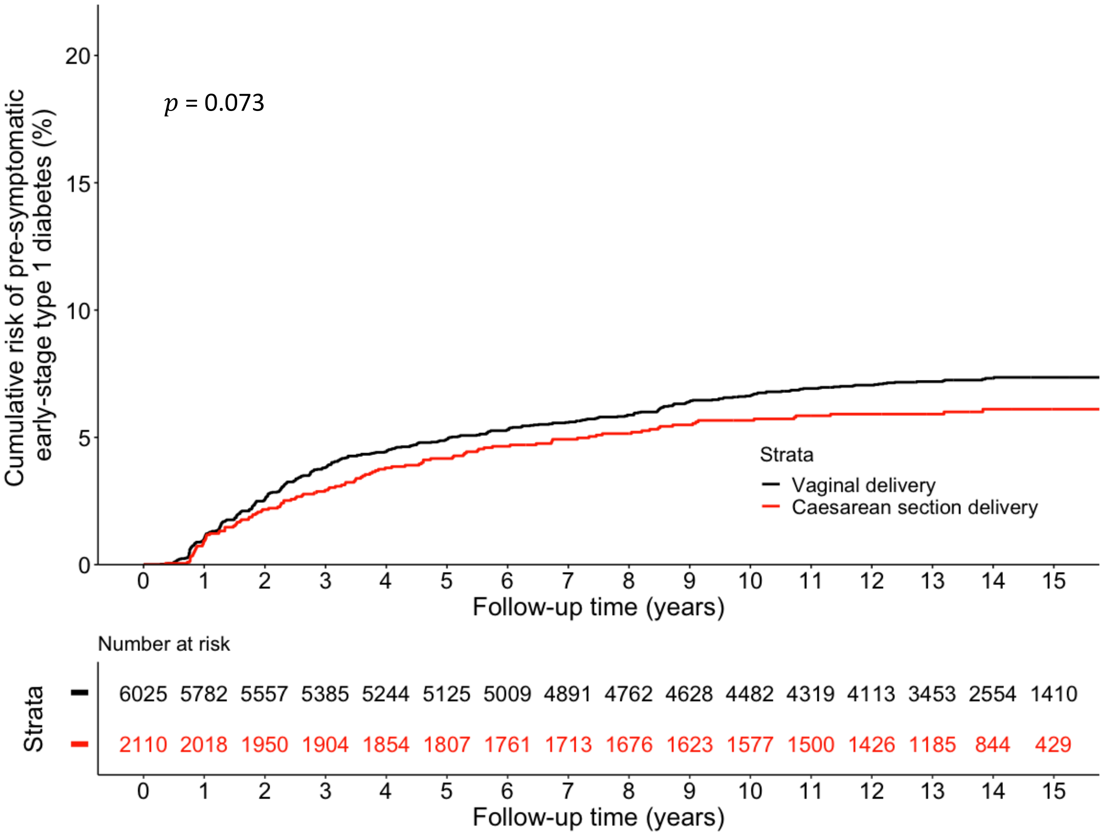

ESM Figure 2: Multivariate Cox proportional hazards model for risk the progression to stage 3 type 1 diabetes from seroconversion to islet autoimmunity in children with pre-symptomatic early-stage type 1 diabetes (multiple islet autoantibodies) born by a mother without type 1 diabetes and who were born full term and as a singleton. a) Whole population, b) European population

a

Covariate	Number, %	Adjusted HR	95%CI	*p* value
Caesarean section	95, 20.9	1.44	(1.07, 1.94)	0.01
USA	158, 34.9	1.08	(0.83, 1.41)	0.55
Maternal age > 28 & ≤ 33 years	175, 38.6	0.82	(0.61, 1.11)	0.2
Maternal age > 33 years	154, 33.9	0.95	(0.71, 1.29)	0.76
Birth weight adjusted by gestational age	221, 48.8	1.32	(1.00, 1.76)	0.05
Female	198, 43.7	1.44	(1.13, 1.83)	0.002
HLA DR3/4	246, 54.3	1.12	(0.88, 1.42)	0.35

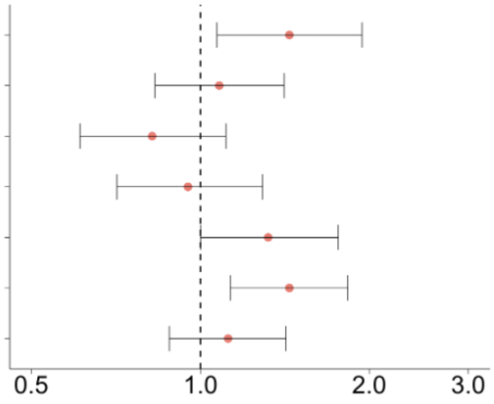

b

Covariate	Number, %	Adjusted HR	95%CI	*p* value
Caesarean section	40, 13.6	2.06	(1.39, 3.07)	0.0003
Maternal age > 28 & ≤ 33 years	124, 42.1	0.77	(0.53, 1.12)	0.18
Maternal age > 33 years	89, 30.2	1.03	(0.71, 1.50)	0.88
Birth weight adjusted by gestational age	147, 49.8	1.12	(0.76, 1.65)	0.58
Female	129, 43.7	1.44	(1.07, 1.95)	0.02
HLA DR3/4	149, 50.5	1.01	(0.75, 1.36)	0.94

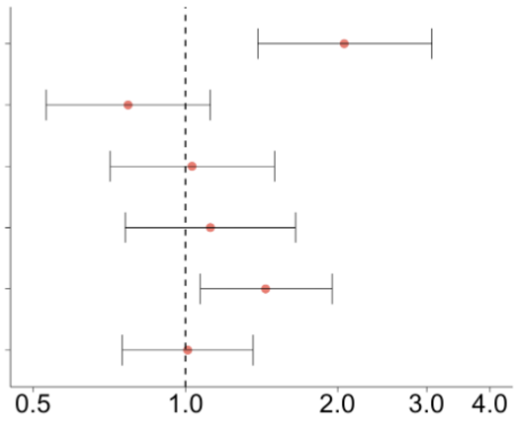

ESM Figure 3: Interactions between Caesarean section and type 1 diabetes susceptibility genes. Kaplan-Meier survival curves for risk of progression to stage 3 type 1 diabetes from seroconversion to islet autoimmunity in children with pre-symptomatic early-stage type 1 diabetes (multiple islet autoantibodies) according to delivery mode stratified for *IFIH1* (a), *MIR3681HG* (b), *CTSH* (c) and *TNFAIP3* (d) genotypes. Type 1 diabetes development curves for Caesarean section and vaginal delivery are shown as red or orange line and black or blue line, respectively, and children with the type 1 diabetes susceptible genotypes are represented in dashed lines and with nonsusceptible genotypes in the solid lines. Numbers under the abscissa are the number of children still followed at each time point.

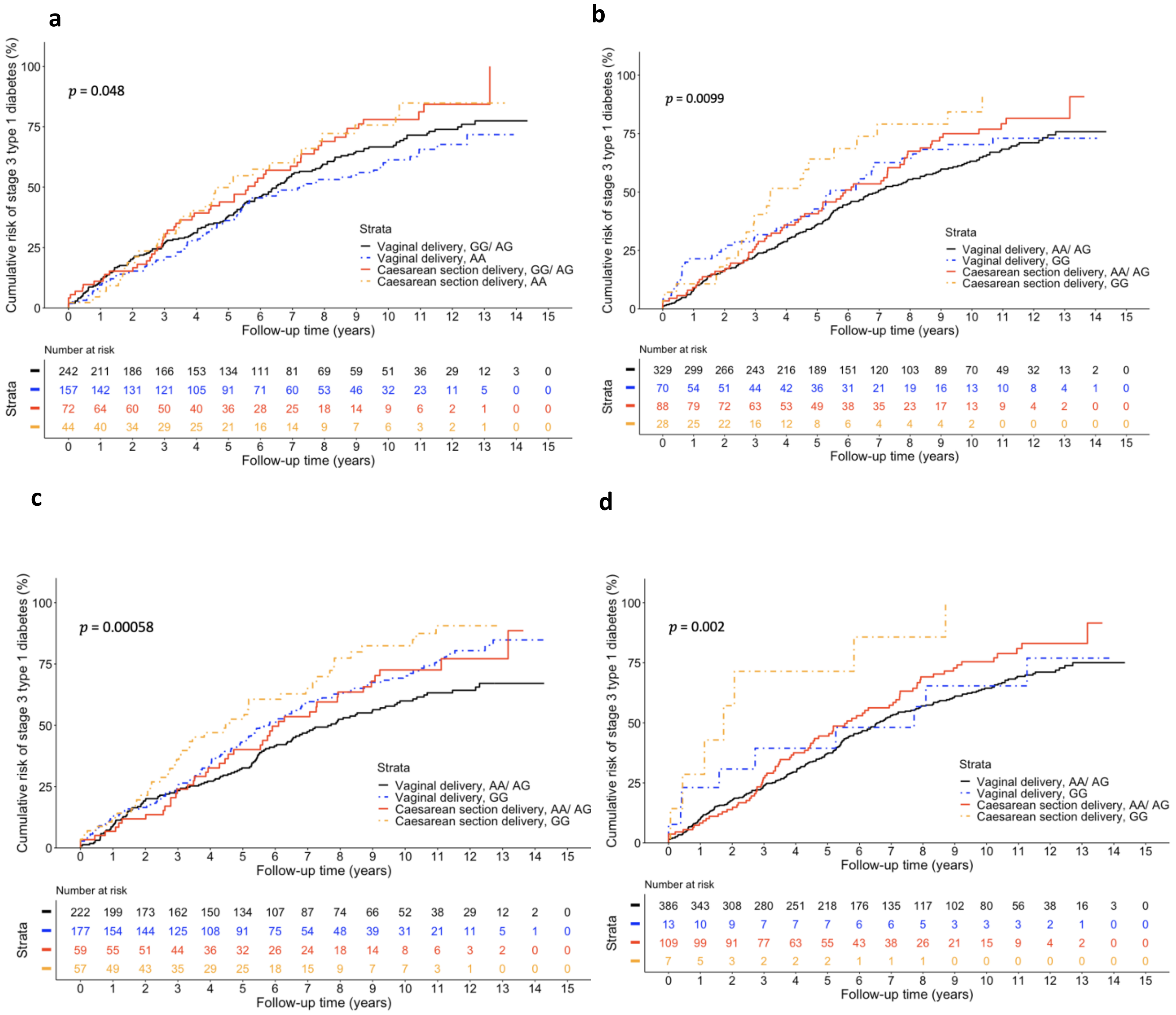

The TEDDY Study Group

Colorado Clinical Center: Marian Rewers, M.D., Ph.D., PI[1,4,6,9,10], Kimberly Bautista[11], Judith Baxter[8,911], Daniel Felipe-Morales, Brigitte I. Frohnert, M.D., Ph.D.[2,13], Marisa Stahl, M.D.[12], Isabel Flores Garcia, Patricia Gesualdo[2,6,11,13], Sierra Hays, Michelle Hoffman[11,12,13], Randi Johnson, Ph.D.[2,3], Rachel Karban[11], Edwin Liu, M.D.[12], Leila Loaiza, Jill Norris, Ph.D.[2,3,11], Holly O'Donnell, Ph.D.[8], Andrea Steck, M.D.[3,13], Kathleen Waugh[6,7,11]. University of Colorado, Anschutz Medical Campus, Barbara Davis Center for Childhood Diabetes, Aurora, CO, USA.

Finland Clinical Center: Jorma Toppari, M.D., Ph.D., PI[¥^1,4,10,13], Olli G. Simell, M.D., Ph.D., Annika Adamsson, Ph.D.[^11], Suvi Ahonen[*±§], Mari Åkerlund[*±§], Sirpa Anttila[μ¤], Leena Hakola, Ph.D.[*±], Sanni Heikura[μ¤], Tiia Honkanen[μ¤], Heikki Hyöty, M.D., Ph.D.[*±6], Jorma Ilonen, M.D., Ph.D.[¥3], Saori Itoshima, M.D.[¥^], Minna Jokipolvi[*±], Sanna Jokipuu[^], Taru Karjalainen[μ¤], Leena Karlsson[^], Jukka Kero, M.D., Ph.D.[¥^3, 13], Marika Korpela[μ¤], Jaakko J. Koskenniemi M.D., Ph.D.[¥^], Miia Kähönen[μ¤11,13], Mikael Knip, M.D., Ph.D.[*±], Minna-Liisa Koivikko[μ¤], Katja Kokkonen[*±], Merja Koskinen[*±], Mirva Koreasalo[*±§2], Kalle Kurppa, M.D., Ph.D.[*±12], Salla Kuusela, M.D. [μ¤], Jarita Kytölä[*±], Mia Laakso[μ¤], Jutta Laiho, Ph.D.[*6], Tiina Latva-aho[μ¤], Siiri Leisku[*±], Laura Leppänen[^], Katri Lindfors, Ph.D.[*12], Maria Lönnrot, M.D., Ph.D.[*±6], Elina Mäntymäki[^], Markus Mattila, Ph.D.[*±2], Maija E. Miettinen, Ph.D.[§2], Tiina Niininen[±*11], Sari Niinistö, Ph.D.[§2], Noora Nurminen[*±], Sami Oikarinen, Ph.D.[*±6], Hanna-Leena Oinas[*±], Paula Ollikainen[μ¤], Zhian Othmani[¥], Sirpa Pohjola [μ¤], Jenna Rautanen[§], Minna Romo[^], Juulia Rönkä[μ¤], Nelli Rönkä[μ¤], Satu Simell, M.D., Ph.D.[¥12], Aino Tihinen[μ¤], Päivi Tossavainen, M.D.[μ¤], Erika Turtinen[μ¤], Mari Vähä-Mäkilä[¥], Eeva Varjonen[^11], Riitta Veijola, M.D., Ph.D.[μ¤13], Irene Viinikangas[μ¤], Suvi M. Virtanen, M.D., Ph.D.[*±§2]. [¥]University of Turku, Turku, Finland, *Tampere University, Tampere, Finland, [μ]University of Oulu, Oulu, Finland, [^]Turku University Hospital, Wellbeing Services County of Southwest Finland, Turku, Finland, [±]Tampere University Hospital, Wellbeing Services County of Pirkanmaa, Tampere, Finland, [¤]Oulu University Hospital, Wellbeing Services County of North Ostrobothia, Oulu, Finland, [§]Finnish Institute for Health and Welfare, Helsinki, Finland.

Georgia/Florida Clinical Center: Richard McIndoe, Ph.D., PI[^4,10], Desmond Schatz, M.D.[*4,7,8], Diane Hopkins[^11], Michael Haller, M.D.[*13], Melissa Gardiner[^11], Ashok Sharma, Ph.D.[^], Laura Jacobsen, M.D.[*13], Percy Gordon^, Jennifer Hosford[^], Sharon Maina[^]. [^]Center for Biotechnology and Genomic Medicine, Augusta University, Augusta, GA, USA. *University of Florida, Pediatric Endocrinology, Gainesville, FL, USA.

Germany Clinical Center: Anette G. Ziegler, M.D., PI[1,3,4,10], Ezio Bonifacio Ph.D.*, Cigdem Sanverdi, Willi Grätz, Anja Heublein, Sandra Hummel, Ph.D.[2], Annette Knopff[7], Melanie Köger, Sibylle Koletzko, M.D.[¶12], Claudia Ramminger[11], Roswith Roth, Ph.D.[8], Jennifer Schmidt, Marlon Scholz, Joanna Stock[8,11,13], Katharina Warncke, M.D.[13], Lorena Müller, Christiane Winkler, Ph.D.[2,11]. Forschergruppe Diabetes e.V. and Institute of Diabetes Research, Helmholtz Zentrum München, Forschergruppe Diabetes, and Klinikum rechts der Isar, Technische Universität München, Neuherberg, Germany. *Center for Regenerative Therapies, TU Dresden, Dresden, Germany, [¶]Dr. von Hauner Children's Hospital, Department of Gastroenterology, Ludwig Maximillians University Munich, Munich, Germany.

Sweden Clinical Center: Åke Lernmark, Ph.D., PI[1,3,4,5,6,8,9,10], Daniel Agardh, M.D., Ph.D.[6,12], Carin Andrén Aronsson, Ph.D.[2,11,12], Rasmus Bennet, Corrado Cilio, Ph.D., M.D.[6], Susanne Dahlberg, Malin Goldman Tsubarah, Emelie Ericson-Hallström, Lina Fransson, Emina Halilovic, Susanne Hyberg, Berglind Jonsdottir, M.D., Ph.D.[11], Naghmeh Karimi, Helena Elding Larsson, M.D., Ph.D.[6,13], Marielle Lindström, Markus Lundgren, M.D., Ph.D.[13], Marlena Maziarz, Ph.D., Jessica Melin, Ph.D.[11], Kobra Rahmati, Anita Ramelius, Falastin Salami, Ph.D., Anette Sjöberg, Evelyn Tekum Amboh, Carina Törn, Ph.D.[3], Ulrika Ulvenhag, Terese Wiktorsson, Åsa Wimar[13]. Lund University, Lund, Sweden.

Washington Clinical Center: William A. Hagopian, M.D., Ph.D., PI[1,3,4,6,7,10,12,13], Michael Killian[6,7,11,12], Claire Cowen Crouch[11,13], Jennifer Skidmore[2], Trevor Bender, Megan Llewellyn, Cody McCall, Arlene Meyer, Jocelyn Meyer, Denise Mulenga[11], Nole Powell, Jared Radtke, Shreya Roy, Preston Tucker. Pacific Northwest Research Institute, Seattle, WA, USA.

Pennsylvania Satellite Center: Dorothy Becker, M.D., Margaret Franciscus, MaryEllen Dalmagro-Elias Smith[2], Ashi Daftary, M.D., Mary Beth Klein, Chrystal Yates. Children's Hospital of Pittsburgh of UPMC, Pittsburgh, PA, USA.

Data Coordinating Center: Jeffrey P. Krischer, Ph.D., PI[1,4,5,9,10], Rajesh Adusumali, Sarah Austin-Gonzalez, Maryouri Avendano, Sandra Baethke, Brant Burkhardt, Ph.D.[6], Martha Butterworth[2], Nicholas Cadigan, Joanna Clasen, Ph.D., Kevin Counts, Laura Gandolfo, Jennifer Garmeson, Veena Gowda, Shu Liu, Xiang Liu, Ph.D.[2,3,8,13], Kristian Lynch, Ph.D. [6,8], Jamie Malloy, Lazarus Mramba, Ph.D.[2], Cristina McCarthy[11], Hemang M. Parikh, Ph.D.[3,8], Cassandra Remedios, Chris Shaffer, Susan Smith[11], Noah Sulman, Ph.D., Roy Tamura, Ph.D.[1,2,11,12,13], Dena Tewey, Henri Thuma, Michael Toth, Ulla Uusitalo, Ph.D.[2], Kendra Vehik, Ph.D.[4,5,6,8,13], Ponni Vijayakandipan, Melissa Wroble, Jimin Yang, Ph.D., R.D.[2], Kenneth Young, Ph.D. *Past staff: Michael Abbondondolo, Lori Ballard, Rasheedah Brown, David Cuthbertson, Stephen Dankyi, Christopher Eberhard, Steven Fiske, David Hadley, Ph.D., Kathleen Heyman, Belinda Hsiao, Christina Karges, Francisco Perez Laras, Hye-Seung Lee, Ph.D., Qian Li, Ph.D., Colleen Maguire, Wendy McLeod, Aubrie Merrell, Steven Meulemans, Jose Moreno, Ryan Quigley, Laura Smith, Ph.D.* University of South Florida, Tampa, FL, USA.

Autoantibody Reference Laboratories: Liping Yu, M.D.^[5], Dongmei Miao, M.D.^, Kathleen Gillespie*[5], Kyla Chandler*, Olivia Pearce*, Sarah Stollery*, Elinor Balch*, Hanah Batholomew*, Zahra Hashmi*. ^Barbara Davis Center for Childhood Diabetes, University of Colorado Denver, *Bristol Medical School, University of Bristol, UK.

Genetics Laboratory: Stephen S. Rich, Ph.D.[3], Wei-Min Chen, Ph.D.[3], Suna Onengut-Gumuscu, Ph.D.[3], Emily Farber, Rebecca Roche Pickin, Ph.D., Jonathan Davis, Jordan Davis, Dan Gallo, Jessica Bonnie, Paul Campolieto. Center for Public Health Genomics, University of Virginia, Charlottesville, VA, USA.

Repository: Chris Deigan. NIDDK Biosample Repository at Fisher BioServices, Rockville, MD, USA. (Previously Ricky Schrock, Polina Malone, Sandra Ke, Niveen Mulholland, Ph.D.)

Project scientist: Beena Akolkar, Ph.D.[1,3,4,5,6,7,9,10]. National Institutes of Diabetes and Digestive and Kidney Diseases, Bethesda, MD, USA.

Other contributors: Thomas Briese, Ph.D.[6], Columbia University, New York, NY, USA. Todd Brusko, Ph.D.[5], University of Florida, Gainesville, FL, USA. Teresa Buckner, Ph.D.[2], University of Northern Colorado, Greeley, CO, USA. Suzanne Bennett Johnson, Ph.D.[8,11], Florida State University, Tallahassee, FL, USA. Eoin McKinney, Ph.D.[5], University of Cambridge, Cambridge, UK. Tomi Pastinen, M.D., Ph.D.[5], The Children's Mercy Hospital, Kansas City, MO, USA. Steffen Ullitz Thorsen, M.D., Ph.D.[2], Department of Clinical Immunology, University of Copenhagen, Copenhagen, Denmark, and Department of Pediatrics and Adolescents, Copenhagen University Hospital, Herlev, Denmark. Eric Triplett, Ph.D.[6], University of Florida, Gainesville, FL, USA.

Committees:

[1]Ancillary Studies, [2]Diet, [3]Genetics, [4]Human Subjects/Publicity/Publications, [5]Immune Markers, [6]Infectious Agents, [7]Laboratory Implementation, [8]Psychosocial, [9]Quality Assurance, [10]Steering, [11]Study Coordinators, [12]Celiac Disease, [13]Clinical Implementation.
